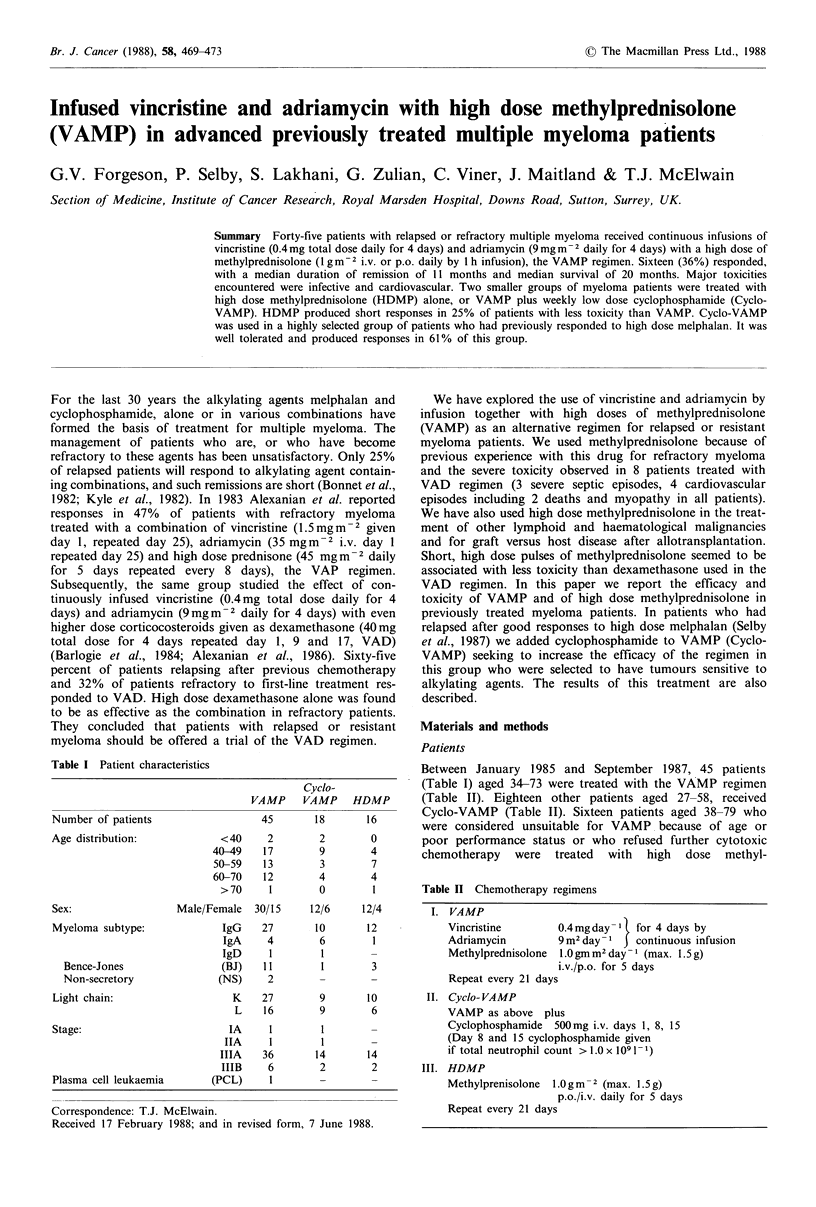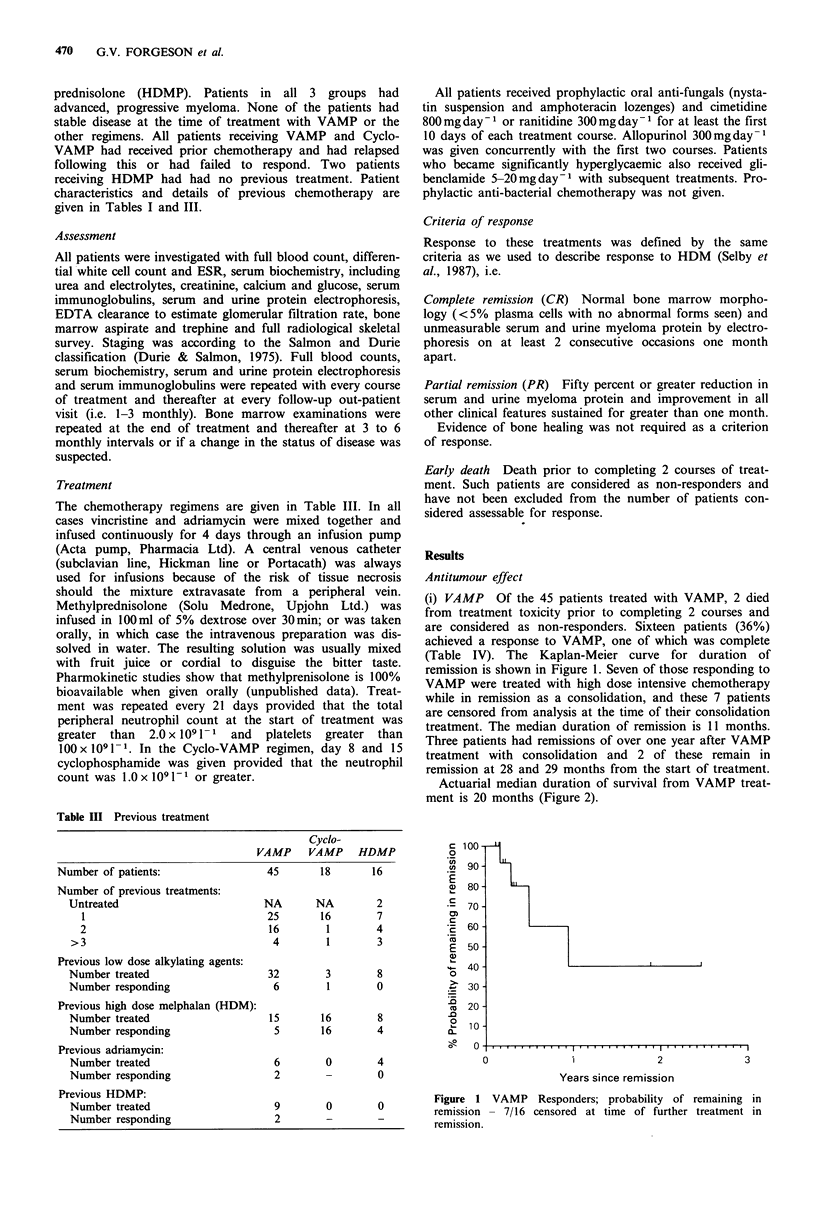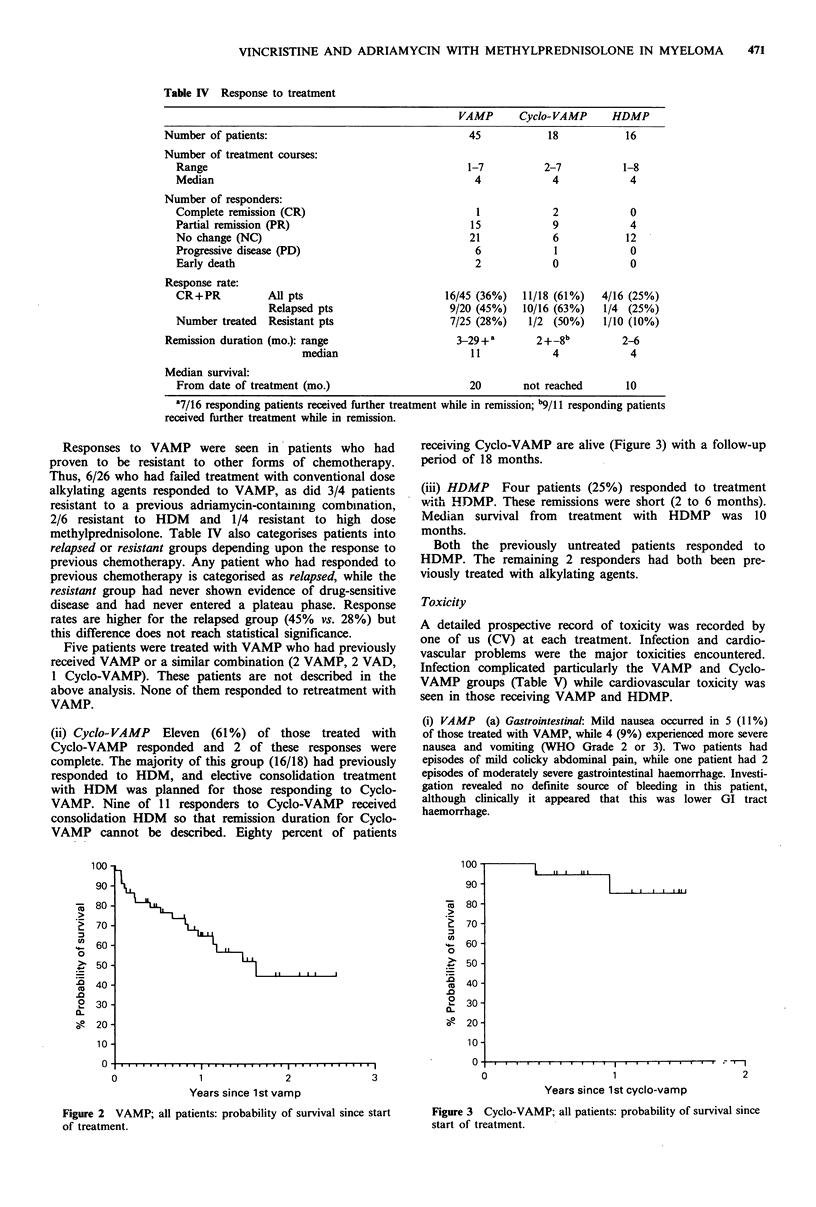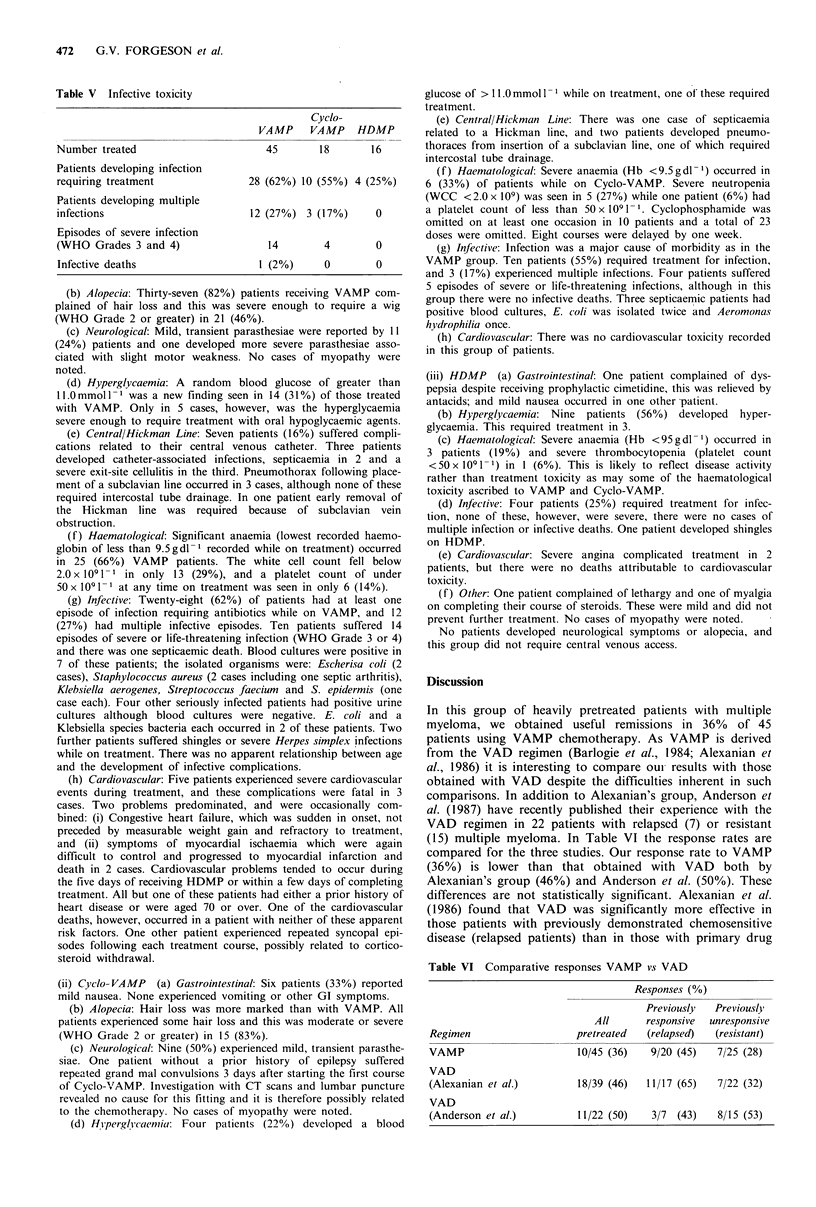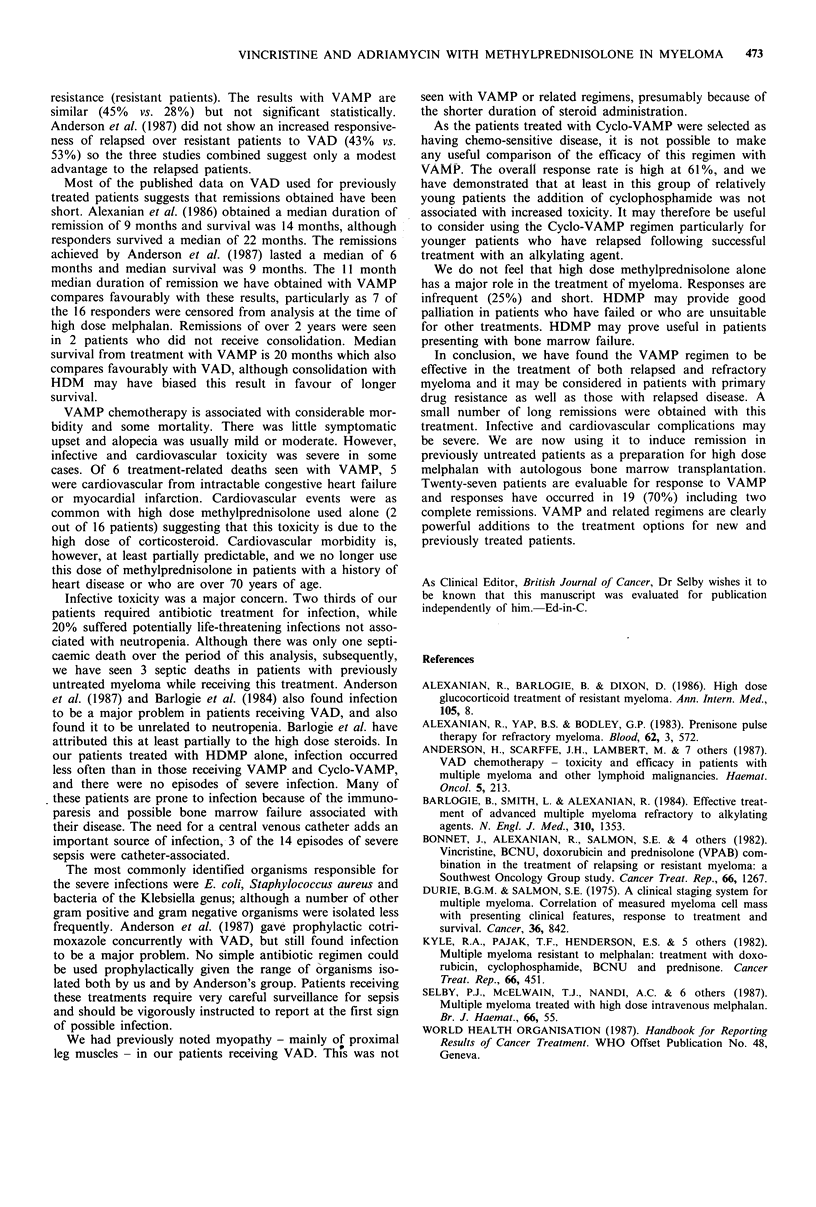# Infused vincristine and adriamycin with high dose methylprednisolone (VAMP) in advanced previously treated multiple myeloma patients.

**DOI:** 10.1038/bjc.1988.243

**Published:** 1988-10

**Authors:** G. V. Forgeson, P. Selby, S. Lakhani, G. Zulian, C. Viner, J. Maitland, T. J. McElwain

**Affiliations:** Institute of Cancer Research, Royal Marsden Hospital, Surrey, UK.

## Abstract

Forty-five patients with relapsed or refractory multiple myeloma received continuous infusions of vincristine (0.4 mg total dose daily for 4 days) and adriamycin (9 mg m-2 daily for 4 days) with a high dose of methylprednisolone (1 g m-2 i.v. or p.o. daily by 1 h infusion), the VAMP regimen. Sixteen (36%) responded, with a median duration of remission of 11 months and median survival of 20 months. Major toxicities encountered were infective and cardiovascular. Two smaller groups of myeloma patients were treated with high dose methylprednisolone (HDMP) alone, or VAMP plus weekly low dose cyclophosphamide (Cyclo-VAMP). HDMP produced short responses in 25% of patients with less toxicity than VAMP. Cyclo-VAMP was used in a highly selected group of patients who had previously responded to high dose melphalan. It was well tolerated and produced responses in 61% of this group.


Br. J. Cancer (1988), 58, 469–473 © The Macmillan Press Ltd., 1988

### Infused vincristine and adriamycin with high dose methylprednisolone (VAMP) in advanced previously treated multiple myeloma patients

G.V. Forgeson, P. Selby, S. Lakhani, G. Zulian, C. Viner, J. Maitland & T.J. McElwain

*Section of Medicine, Institute of Cancer Research, Royal Marsden Hospital, Downs Road, Sutton, Surrey, UK.*

**Summary** Forty-five patients with relapsed or refractory multiple myeloma received continuous infusions of vincristine (0.4 mg total dose daily for 4 days) and adriamycin (9 mg m$^{-2}$ daily for 4 days) with a high dose of methylprednisolone (1 g m$^{-2}$ i.v. or p.o. daily by 1 h infusion), the VAMP regimen. Sixteen (36%) responded, with a median duration of remission of 11 months and median survival of 20 months. Major toxicities encountered were infective and cardiovascular. Two smaller groups of myeloma patients were treated with high dose methylprednisolone (HDMP) alone, or VAMP plus weekly low dose cyclophosphamide (Cyclo-VAMP). HDMP produced short responses in 25% of patients with less toxicity than VAMP. Cyclo-VAMP was used in a highly selected group of patients who had previously responded to high dose melphalan. It was well tolerated and produced responses in 61% of this group.

For the last 30 years the alkylating agents melphalan and cyclophosphamide, alone or in various combinations have formed the basis of treatment for multiple myeloma. The management of patients who are, or who have become refractory to these agents has been unsatisfactory. Only 25% of relapsed patients will respond to alkylating agent containing combinations, and such remissions are short (Bonnet *et al.*, 1982; Kyle *et al.*, 1982). In 1983 Alexanian *et al.* reported responses in 47% of patients with refractory myeloma treated with a combination of vincristine (1.5 mg m$^{-2}$ given day 1, repeated day 25), adriamycin (35 mg m$^{-2}$ i.v. day 1 repeated day 25) and high dose prednisone (45 mg m$^{-2}$ daily for 5 days repeated every 8 days), the VAP regimen. Subsequently, the same group studied the effect of continuously infused vincristine (0.4 mg total dose daily for 4 days) and adriamycin (9 mg m$^{-2}$ daily for 4 days) with even higher dose corticocosteroids given as dexamethasone (40 mg total dose for 4 days repeated day 1, 9 and 17, VAD) (Barlogie *et al.*, 1984; Alexanian *et al.*, 1986). Sixty-five percent of patients relapsing after previous chemotherapy and 32% of patients refractory to first-line treatment responded to VAD. High dose dexamethasone alone was found to be as effective as the combination in refractory patients. They concluded that patients with relapsed or resistant myeloma should be offered a trial of the VAD regimen.

**Table I** Patient characteristics

| | | *VAMP* | *Cyclo-VAMP* | *HDMP* |
|---|---|---|---|---|
| Number of patients | | 45 | 18 | 16 |
| Age distribution: | <40 | 2 | 2 | 0 |
| | 40–49 | 17 | 9 | 4 |
| | 50–59 | 13 | 3 | 7 |
| | 60–70 | 12 | 4 | 4 |
| | >70 | 1 | 0 | 1 |
| Sex: | Male/Female | 30/15 | 12/6 | 12/4 |
| Myeloma subtype: | IgG | 27 | 10 | 12 |
| | IgA | 4 | 6 | 1 |
| | IgD | 1 | 1 | – |
| Bence-Jones | (BJ) | 11 | 1 | 3 |
| Non-secretory | (NS) | 2 | – | – |
| Light chain: | K | 27 | 9 | 10 |
| | L | 16 | 9 | 6 |
| Stage: | IA | 1 | 1 | – |
| | IIA | 1 | 1 | – |
| | IIIA | 36 | 14 | 14 |
| | IIIB | 6 | 2 | 2 |
| Plasma cell leukaemia | (PCL) | 1 | – | – |

Correspondence: T.J. McElwain.
Received 17 February 1988; and in revised form, 7 June 1988.

We have explored the use of vincristine and adriamycin by infusion together with high doses of methylprednisolone (VAMP) as an alternative regimen for relapsed or resistant myeloma patients. We used methylprednisolone because of previous experience with this drug for refractory myeloma and the severe toxicity observed in 8 patients treated with VAD regimen (3 severe septic episodes, 4 cardiovascular episodes including 2 deaths and myopathy in all patients). We have also used high dose methylprednisolone in the treatment of other lymphoid and haematological malignancies and for graft versus host disease after allotransplantation. Short, high dose pulses of methylprednisolone seemed to be associated with less toxicity than dexamethasone used in the VAD regimen. In this paper we report the efficacy and toxicity of VAMP and of high dose methylprednisolone in previously treated myeloma patients. In patients who had relapsed after good responses to high dose melphalan (Selby *et al.*, 1987) we added cyclophosphamide to VAMP (Cyclo-VAMP) seeking to increase the efficacy of the regimen in this group who were selected to have tumours sensitive to alkylating agents. The results of this treatment are also described.

### Materials and methods

### *Patients*

Between January 1985 and September 1987, 45 patients (Table I) aged 34–73 were treated with the VAMP regimen (Table II). Eighteen other patients aged 27–58, received Cyclo-VAMP (Table II). Sixteen patients aged 38–79 who were considered unsuitable for VAMP because of age or poor performance status or who refused further cytotoxic chemotherapy were treated with high dose methyl-

**Table II** Chemotherapy regimens

I. *VAMP*

Vincristine 0.4 mg day$^{-1}$ } for 4 days by

Adriamycin 9 m$^2$ day$^{-1}$ } continuous infusion

Methylprednisolone 1.0 gm m$^2$ day$^{-1}$ (max. 1.5 g) i.v./p.o. for 5 days

Repeat every 21 days

II. *Cyclo-VAMP*

VAMP as above plus

Cyclophosphamide 500 mg i.v. days 1, 8, 15

(Day 8 and 15 cyclophosphamide given if total neutrophil count >1.0 × 10$^9$ l$^{-1}$)

III. *HDMP*
Methylprenisolone 1.0 g m$^{-2}$ (max. 1.5 g) p.o./i.v. daily for 5 days
Repeat every 21 days prednisolone (HDMP). Patients in all 3 groups had advanced, progressive myeloma. None of the patients had stable disease at the time of treatment with VAMP or the other regimens. All patients receiving VAMP and Cyclo-VAMP had received prior chemotherapy and had relapsed following this or had failed to respond. Two patients receiving HDMP had had no previous treatment. Patient characteristics and details of previous chemotherapy are given in Tables I and III.

*Assessment*

All patients were investigated with full blood count, differential white cell count and ESR, serum biochemistry, including urea and electrolytes, creatinine, calcium and glucose, serum immunoglobulins, serum and urine protein electrophoresis, EDTA clearance to estimate glomerular filtration rate, bone marrow aspirate and trephine and full radiological skeletal survey. Staging was according to the Salmon and Durie classification (Durie & Salmon, 1975). Full blood counts, serum biochemistry, serum and urine protein electrophoresis and serum immunoglobulins were repeated with every course of treatment and thereafter at every follow-up out-patient visit (i.e. 1–3 monthly). Bone marrow examinations were repeated at the end of treatment and thereafter at 3 to 6 monthly intervals or if a change in the status of disease was suspected.

*Treatment*

The chemotherapy regimens are given in Table III. In all cases vincristine and adriamycin were mixed together and infused continuously for 4 days through an infusion pump (Acta pump, Pharmacia Ltd). A central venous catheter (subclavian line, Hickman line or Portacath) was always used for infusions because of the risk of tissue necrosis should the mixture extravasate from a peripheral vein. Methylprednisolone (Solu Medrone, Upjohn Ltd.) was infused in 100 ml of 5% dextrose over 30 min; or was taken orally, in which case the intravenous preparation was dissolved in water. The resulting solution was usually mixed with fruit juice or cordial to disguise the bitter taste. Pharmokinetic studies show that methylprenisolone is 100% bioavailable when given orally (unpublished data). Treatment was repeated every 21 days provided that the total peripheral neutrophil count at the start of treatment was greater than $2.0 \times 10^9 l^{-1}$ and platelets greater than $100 \times 10^9 l^{-1}$. In the Cyclo-VAMP regimen, day 8 and 15 cyclophosphamide was given provided that the neutrophil count was $1.0 \times 10^9 l^{-1}$ or greater.

**Table III** Previous treatment

| | VAMP | Cyclo-VAMP | HDMP |
|---|---|---|---|
| Number of patients: | 45 | 18 | 16 |
| Number of previous treatments: | | | |
| Untreated | NA | NA | 2 |
| 1 | 25 | 16 | 7 |
| 2 | 16 | 1 | 4 |
| >3 | 4 | 1 | 3 |
| Previous low dose alkylating agents: | | | |
| Number treated | 32 | 3 | 8 |
| Number responding | 6 | 1 | 0 |
| Previous high dose melphalan (HDM): | | | |
| Number treated | 15 | 16 | 8 |
| Number responding | 5 | 16 | 4 |
| Previous adriamycin: | | | |
| Number treated | 6 | 0 | 4 |
| Number responding | 2 | – | 0 |
| Previous HDMP: | | | |
| Number treated | 9 | 0 | 0 |
| Number responding | 2 | – | – |

All patients received prophylactic oral anti-fungals (nystatin suspension and amphoteracin lozenges) and cimetidine 800 mg day$^{-1}$ or ranitidine 300 mg day$^{-1}$ for at least the first 10 days of each treatment course. Allopurinol 300 mg day$^{-1}$ was given concurrently with the first two courses. Patients who became significantly hyperglycaemic also received glibenclamide 5–20 mg day$^{-1}$ with subsequent treatments. Prophylactic anti-bacterial chemotherapy was not given.

*Criteria of response*

Response to these treatments was defined by the same criteria as we used to describe response to HDM (Selby *et al.*, 1987), i.e.

*Complete remission* (*CR*) Normal bone marrow morphology (<5% plasma cells with no abnormal forms seen) and unmeasurable serum and urine myeloma protein by electrophoresis on at least 2 consecutive occasions one month apart.

*Partial remission* (*PR*) Fifty percent or greater reduction in serum and urine myeloma protein and improvement in all other clinical features sustained for greater than one month.

Evidence of bone healing was not required as a criterion of response.

*Early death* Death prior to completing 2 courses of treatment. Such patients are considered as non-responders and have not been excluded from the number of patients considered assessable for response.

### Results

*Antitumour effect*

(i) *VAMP* Of the 45 patients treated with VAMP, 2 died from treatment toxicity prior to completing 2 courses and are considered as non-responders. Sixteen patients (36%) achieved a response to VAMP, one of which was complete (Table IV). The Kaplan-Meier curve for duration of remission is shown in Figure 1. Seven of those responding to VAMP were treated with high dose intensive chemotherapy while in remission as a consolidation, and these 7 patients are censored from analysis at the time of their consolidation treatment. The median duration of remission is 11 months. Three patients had remissions of over one year after VAMP treatment with consolidation and 2 of these remain in remission at 28 and 29 months from the start of treatment.

Actuarial median duration of survival from VAMP treatment is 20 months (Figure 2).

**Figure 1** VAMP Responders; probability of remaining in remission – 7/16 censored at time of further treatment in remission.

**Table IV** Response to treatment

| | | VAMP | Cyclo-VAMP | HDMP |
|---|---|---|---|---|
| Number of patients: | | 45 | 18 | 16 |
| Number of treatment courses: | | | | |
| Range | | 1–7 | 2–7 | 1–8 |
| Median | | 4 | 4 | 4 |
| Number of responders: | | | | |
| Complete remission (CR) | | 1 | 2 | 0 |
| Partial remission (PR) | | 15 | 9 | 4 |
| No change (NC) | | 21 | 6 | 12 |
| Progressive disease (PD) | | 6 | 1 | 0 |
| Early death | | 2 | 0 | 0 |
| Response rate: | | | | |
| CR+PR | All pts | 16/45 (36%) | 11/18 (61%) | 4/16 (25%) |
| | Relapsed pts | 9/20 (45%) | 10/16 (63%) | 1/4 (25%) |
| Number treated | Resistant pts | 7/25 (28%) | 1/2 (50%) | 1/10 (10%) |
| Remission duration (mo.): range | | 3–29+[a] | 2+–8[b] | 2–6 |
| median | | 11 | 4 | 4 |
| Median survival: | | | | |
| From date of treatment (mo.) | | 20 | not reached | 10 |

[a]7/16 responding patients received further treatment while in remission; [b]9/11 responding patients received further treatment while in remission.

Responses to VAMP were seen in patients who had proven to be resistant to other forms of chemotherapy. Thus, 6/26 who had failed treatment with conventional dose alkylating agents responded to VAMP, as did 3/4 patients resistant to a previous adriamycin-containing combination, 2/6 resistant to HDM and 1/4 resistant to high dose methylprednisolone. Table IV also categorises patients into *relapsed* or *resistant* groups depending upon the response to previous chemotherapy. Any patient who had responded to previous chemotherapy is categorised as *relapsed*, while the *resistant* group had never shown evidence of drug-sensitive disease and had never entered a plateau phase. Response rates are higher for the relapsed group (45% *vs.* 28%) but this difference does not reach statistical significance.

Five patients were treated with VAMP who had previously received VAMP or a similar combination (2 VAMP, 2 VAD, 1 Cyclo-VAMP). These patients are not described in the above analysis. None of them responded to retreatment with VAMP.

(ii) *Cyclo-VAMP* Eleven (61%) of those treated with Cyclo-VAMP responded and 2 of these responses were complete. The majority of this group (16/18) had previously responded to HDM, and elective consolidation treatment with HDM was planned for those responding to Cyclo-VAMP. Nine of 11 responders to Cyclo-VAMP received consolidation HDM so that remission duration for Cyclo-VAMP cannot be described. Eighty percent of patients receiving Cyclo-VAMP are alive (Figure 3) with a follow-up period of 18 months.

(iii) *HDMP* Four patients (25%) responded to treatment with HDMP. These remissions were short (2 to 6 months). Median survival from treatment with HDMP was 10 months.

Both the previously untreated patients responded to HDMP. The remaining 2 responders had both been previously treated with alkylating agents.

*Toxicity*

A detailed prospective record of toxicity was recorded by one of us (CV) at each treatment. Infection and cardiovascular problems were the major toxicities encountered. Infection complicated particularly the VAMP and Cyclo-VAMP groups (Table V) while cardiovascular toxicity was seen in those receiving VAMP and HDMP.

(i) *VAMP* (a) *Gastrointestinal*: Mild nausea occurred in 5 (11%) of those treated with VAMP, while 4 (9%) experienced more severe nausea and vomiting (WHO Grade 2 or 3). Two patients had episodes of mild colicky abdominal pain, while one patient had 2 episodes of moderately severe gastrointestinal haemorrhage. Investigation revealed no definite source of bleeding in this patient, although clinically it appeared that this was lower GI tract haemorrhage.

**Figure 2** VAMP; all patients: probability of survival since start of treatment.

**Figure 3** Cyclo-VAMP; all patients: probability of survival since start of treatment.

**Table V** Infective toxicity

| | VAMP | Cyclo-VAMP | HDMP |
|---|---|---|---|
| Number treated | 45 | 18 | 16 |
| Patients developing infection requiring treatment | 28 (62%) | 10 (55%) | 4 (25%) |
| Patients developing multiple infections | 12 (27%) | 3 (17%) | 0 |
| Episodes of severe infection (WHO Grades 3 and 4) | 14 | 4 | 0 |
| Infective deaths | 1 (2%) | 0 | 0 |

(b) *Alopecia*: Thirty-seven (82%) patients receiving VAMP complained of hair loss and this was severe enough to require a wig (WHO Grade 2 or greater) in 21 (46%).

(c) *Neurological*: Mild, transient parasthesiae were reported by 11 (24%) patients and one developed more severe parasthesiae associated with slight motor weakness. No cases of myopathy were noted.

(d) *Hyperglycaemia*: A random blood glucose of greater than 11.0 mmol l$^{-1}$ was a new finding seen in 14 (31%) of those treated with VAMP. Only in 5 cases, however, was the hyperglycaemia severe enough to require treatment with oral hypoglycaemic agents.

(e) *Central/Hickman Line*: Seven patients (16%) suffered complications related to their central venous catheter. Three patients developed catheter-associated infections, septicaemia in 2 and a severe exit-site cellulitis in the third. Pneumothorax following placement of a subclavian line occurred in 3 cases, although none of these required intercostal tube drainage. In one patient early removal of the Hickman line was required because of subclavian vein obstruction.

(f) *Haematological*: Significant anaemia (lowest recorded haemoglobin of less than 9.5 g dl$^{-1}$ recorded while on treatment) occurred in 25 (66%) VAMP patients. The white cell count fell below $2.0 \times 10^9$ l$^{-1}$ in only 13 (29%), and a platelet count of under $50 \times 10^9$ l$^{-1}$ at any time on treatment was seen in only 6 (14%).

(g) *Infective*: Twenty-eight (62%) of patients had at least one episode of infection requiring antibiotics while on VAMP, and 12 (27%) had multiple infective episodes. Ten patients suffered 14 episodes of severe or life-threatening infection (WHO Grade 3 or 4) and there was one septicaemic death. Blood cultures were positive in 7 of these patients; the isolated organisms were: *Escherisa coli* (2 cases), *Staphylococcus aureus* (2 cases including one septic arthritis), *Klebsiella aerogenes*, *Streptococcus faecium* and *S. epidermis* (one case each). Four other seriously infected patients had positive urine cultures although blood cultures were negative. *E. coli* and a Klebsiella species bacteria each occurred in 2 of these patients. Two further patients suffered shingles or severe *Herpes simplex* infections while on treatment. There was no apparent relationship between age and the development of infective complications.

(h) *Cardiovascular*: Five patients experienced severe cardiovascular events during treatment, and these complications were fatal in 3 cases. Two problems predominated, and were occasionally combined: (i) Congestive heart failure, which was sudden in onset, not preceded by measurable weight gain and refractory to treatment, and (ii) symptoms of myocardial ischaemia which were again difficult to control and progressed to myocardial infarction and death in 2 cases. Cardiovascular problems tended to occur during the five days of receiving HDMP or within a few days of completing treatment. All but one of these patients had either a prior history of heart disease or were aged 70 or over. One of the cardiovascular deaths, however, occurred in a patient with neither of these apparent risk factors. One other patient experienced repeated syncopal episodes following each treatment course, possibly related to corticosteroid withdrawal.

(ii) *Cyclo-VAMP* (a) *Gastrointestinal*: Six patients (33%) reported mild nausea. None experienced vomiting or other GI symptoms.

(b) *Alopecia*: Hair loss was more marked than with VAMP. All patients experienced some hair loss and this was moderate or severe (WHO Grade 2 or greater) in 15 (83%).

(c) *Neurological*: Nine (50%) experienced mild, transient parasthesiae. One patient without a prior history of epilepsy suffered repeated grand mal convulsions 3 days after starting the first course of Cyclo-VAMP. Investigation with CT scans and lumbar puncture revealed no cause for this fitting and it is therefore possibly related to the chemotherapy. No cases of myopathy were noted.

(d) *Hyperglycaemia*: Four patients (22%) developed a blood glucose of >11.0 mmol l$^{-1}$ while on treatment, one of these required treatment.

(e) *Central/Hickman Line*: There was one case of septicaemia related to a Hickman line, and two patients developed pneumothoraces from insertion of a subclavian line, one of which required intercostal tube drainage.

(f) *Haematological*: Severe anaemia (Hb <9.5 g dl$^{-1}$) occurred in 6 (33%) of patients while on Cyclo-VAMP. Severe neutropenia (WCC $<2.0 \times 10^9$) was seen in 5 (27%) while one patient (6%) had a platelet count of less than $50 \times 10^9$ l$^{-1}$. Cyclophosphamide was omitted on at least one occasion in 10 patients and a total of 23 doses were omitted. Eight courses were delayed by one week.

(g) *Infective*: Infection was a major cause of morbidity as in the VAMP group. Ten patients (55%) required treatment for infection, and 3 (17%) experienced multiple infections. Four patients suffered 5 episodes of severe or life-threatening infections, although in this group there were no infective deaths. Three septicaemic patients had positive blood cultures, *E. coli* was isolated twice and *Aeromonas hydrophilia* once.

(h) *Cardiovascular*: There was no cardiovascular toxicity recorded in this group of patients.

(iii) *HDMP* (a) *Gastrointestinal*: One patient complained of dyspepsia despite receiving prophylactic cimetidine, this was relieved by antacids; and mild nausea occurred in one other patient.

(b) *Hyperglycaemia*: Nine patients (56%) developed hyperglycaemia. This required treatment in 3.

(c) *Haematological*: Severe anaemia (Hb <95 g dl$^{-1}$) occurred in 3 patients (19%) and severe thrombocytopenia (platelet count $<50 \times 10^9$ l$^{-1}$) in 1 (6%). This is likely to reflect disease activity rather than treatment toxicity as may some of the haematological toxicity ascribed to VAMP and Cyclo-VAMP.

(d) *Infective*: Four patients (25%) required treatment for infection, none of these, however, were severe, there were no cases of multiple infection or infective deaths. One patient developed shingles on HDMP.

(e) *Cardiovascular*: Severe angina complicated treatment in 2 patients, but there were no deaths attributable to cardiovascular toxicity.

(f) *Other*: One patient complained of lethargy and one of myalgia on completing their course of steroids. These were mild and did not prevent further treatment. No cases of myopathy were noted.

No patients developed neurological symptoms or alopecia, and this group did not require central venous access.

### Discussion

In this group of heavily pretreated patients with multiple myeloma, we obtained useful remissions in 36% of 45 patients using VAMP chemotherapy. As VAMP is derived from the VAD regimen (Barlogie *et al.*, 1984; Alexanian *et al.*, 1986) it is interesting to compare our results with those obtained with VAD despite the difficulties inherent in such comparisons. In addition to Alexanian's group, Anderson *et al.* (1987) have recently published their experience with the VAD regimen in 22 patients with relapsed (7) or resistant (15) multiple myeloma. In Table VI the response rates are compared for the three studies. Our response rate to VAMP (36%) is lower than that obtained with VAD both by Alexanian's group (46%) and Anderson *et al.* (50%). These differences are not statistically significant. Alexanian *et al.* (1986) found that VAD was significantly more effective in those patients with previously demonstrated chemosensitive disease (relapsed patients) than in those with primary drug

**Table VI** Comparative responses VAMP *vs* VAD

| | Responses (%) | | |
|---|---|---|---|
| Regimen | All pretreated | Previously responsive (relapsed) | Previously unresponsive (resistant) |
| VAMP | 10/45 (36) | 9/20 (45) | 7/25 (28) |
| VAD (Alexanian *et al.*) | 18/39 (46) | 11/17 (65) | 7/22 (32) |
| VAD (Anderson *et al.*) | 11/22 (50) | 3/7 (43) | 8/15 (53) |

resistance (resistant patients). The results with VAMP are similar (45% *vs.* 28%) but not significant statistically. Anderson *et al.* (1987) did not show an increased responsiveness of relapsed over resistant patients to VAD (43% *vs.* 53%) so the three studies combined suggest only a modest advantage to the relapsed patients.

Most of the published data on VAD used for previously treated patients suggests that remissions obtained have been short. Alexanian *et al.* (1986) obtained a median duration of remission of 9 months and survival was 14 months, although responders survived a median of 22 months. The remissions achieved by Anderson *et al.* (1987) lasted a median of 6 months and median survival was 9 months. The 11 month median duration of remission we have obtained with VAMP compares favourably with these results, particularly as 7 of the 16 responders were censored from analysis at the time of high dose melphalan. Remissions of over 2 years were seen in 2 patients who did not receive consolidation. Median survival from treatment with VAMP is 20 months which also compares favourably with VAD, although consolidation with HDM may have biased this result in favour of longer survival.

VAMP chemotherapy is associated with considerable morbidity and some mortality. There was little symptomatic upset and alopecia was usually mild or moderate. However, infective and cardiovascular toxicity was severe in some cases. Of 6 treatment-related deaths seen with VAMP, 5 were cardiovascular from intractable congestive heart failure or myocardial infarction. Cardiovascular events were as common with high dose methylprednisolone used alone (2 out of 16 patients) suggesting that this toxicity is due to the high dose of corticosteroid. Cardiovascular morbidity is, however, at least partially predictable, and we no longer use this dose of methylprednisolone in patients with a history of heart disease or who are over 70 years of age.

Infective toxicity was a major concern. Two thirds of our patients required antibiotic treatment for infection, while 20% suffered potentially life-threatening infections not associated with neutropenia. Although there was only one septicaemic death over the period of this analysis, subsequently, we have seen 3 septic deaths in patients with previously untreated myeloma while receiving this treatment. Anderson *et al.* (1987) and Barlogie *et al.* (1984) also found infection to be a major problem in patients receiving VAD, and also found it to be unrelated to neutropenia. Barlogie *et al.* have attributed this at least partially to the high dose steroids. In our patients treated with HDMP alone, infection occurred less often than in those receiving VAMP and Cyclo-VAMP, and there were no episodes of severe infection. Many of these patients are prone to infection because of the immunoparesis and possible bone marrow failure associated with their disease. The need for a central venous catheter adds an important source of infection, 3 of the 14 episodes of severe sepsis were catheter-associated.

The most commonly identified organisms responsible for the severe infections were *E. coli*, *Staphylococcus aureus* and bacteria of the Klebsiella genus; although a number of other gram positive and gram negative organisms were isolated less frequently. Anderson *et al.* (1987) gave prophylactic cotrimoxazole concurrently with VAD, but still found infection to be a major problem. No simple antibiotic regimen could be used prophylactically given the range of organisms isolated both by us and by Anderson's group. Patients receiving these treatments require very careful surveillance for sepsis and should be vigorously instructed to report at the first sign of possible infection.

We had previously noted myopathy – mainly of proximal leg muscles – in our patients receiving VAD. This was not seen with VAMP or related regimens, presumably because of the shorter duration of steroid administration.

As the patients treated with Cyclo-VAMP were selected as having chemo-sensitive disease, it is not possible to make any useful comparison of the efficacy of this regimen with VAMP. The overall response rate is high at 61%, and we have demonstrated that at least in this group of relatively young patients the addition of cyclophosphamide was not associated with increased toxicity. It may therefore be useful to consider using the Cyclo-VAMP regimen particularly for younger patients who have relapsed following successful treatment with an alkylating agent.

We do not feel that high dose methylprednisolone alone has a major role in the treatment of myeloma. Responses are infrequent (25%) and short. HDMP may provide good palliation in patients who have failed or who are unsuitable for other treatments. HDMP may prove useful in patients presenting with bone marrow failure.

In conclusion, we have found the VAMP regimen to be effective in the treatment of both relapsed and refractory myeloma and it may be considered in patients with primary drug resistance as well as those with relapsed disease. A small number of long remissions were obtained with this treatment. Infective and cardiovascular complications may be severe. We are now using it to induce remission in previously untreated patients as a preparation for high dose melphalan with autologous bone marrow transplantation. Twenty-seven patients are evaluable for response to VAMP and responses have occurred in 19 (70%) including two complete remissions. VAMP and related regimens are clearly powerful additions to the treatment options for new and previously treated patients.

As Clinical Editor, *British Journal of Cancer*, Dr Selby wishes it to be known that this manuscript was evaluated for publication independently of him.—Ed-in-C.

**References**

ALEXANIAN, R., BARLOGIE, B. & DIXON, D. (1986). High dose glucocorticoid treatment of resistant myeloma. *Ann. Intern. Med.*, **105**, 8.

ALEXANIAN, R., YAP, B.S. & BODLEY, G.P. (1983). Prenisone pulse therapy for refractory myeloma. *Blood*, **62**, 3, 572.

ANDERSON, H., SCARFFE, J.H., LAMBERT, M. & 7 others (1987). VAD chemotherapy – toxicity and efficacy in patients with multiple myeloma and other lymphoid malignancies. *Haemat. Oncol.* **5**, 213.

BARLOGIE, B., SMITH, L. & ALEXANIAN, R. (1984). Effective treatment of advanced multiple myeloma refractory to alkylating agents. *N. Engl. J. Med.*, **310**, 1353.

BONNET, J., ALEXANIAN, R., SALMON, S.E. & 4 others (1982). Vincristine, BCNU, doxorubicin and prednisolone (VPAB) combination in the treatment of relapsing or resistant myeloma: a Southwest Oncology Group study. *Cancer Treat. Rep.*, **66**, 1267.

DURIE, B.G.M. & SALMON, S.E. (1975). A clinical staging system for multiple myeloma. Correlation of measured myeloma cell mass with presenting clinical features, response to treatment and survival. *Cancer*, **36**, 842.

KYLE, R.A., PAJAK, T.F., HENDERSON, E.S. & 5 others (1982). Multiple myeloma resistant to melphalan: treatment with doxorubicin, cyclophosphamide, BCNU and prednisone. *Cancer Treat. Rep.*, **66**, 451.

SELBY, P.J., McELWAIN, T.J., NANDI, A.C. & 6 others (1987). Multiple myeloma treated with high dose intravenous melphalan. *Br. J. Haemat.*, **66**, 55.

WORLD HEALTH ORGANISATION (1987). *Handbook for Reporting Results of Cancer Treatment*. WHO Offset Publication No. 48, Geneva.